# Funomia

**Published:** 1906-01-15

**Authors:** 


### FUNOMIA.

BY TOOTHSOM TOPICS TULLER.

We had a meeting last month with a certain Indian, Dr. John Quince, as essayist.

Of course, every one knows if we had John Quince we had a good paper, and a good fellow (I know this to be so of my own free will and accord), but he is not a good Indian, for he is not dead—not yet.

The paper presented was the result of conclusions drawn from a protracted series of tabulated experiments in fusing dental porcelain.

Of course, there was funomia in it, but we did not know just how much, nor whether chemical or physical, though the Encyclopedia Brittanica, which we know by heart, says: "The making of pottery (porcelain) depends upon the *chemical change* that takes place when clay (*kaolin*) is heated by fire.

The meeting was large, the hall being overcrowded. The discussants of the paper were Drs. John Nye, John Buck, John Nye, John Buck (interrupted by John Quince), John Crux, John Proth, John Buck, John Nye, John Q., closing.

A few others butted in who were not of the symbol John, but the majority did not wish to display their ignorance of chemistry, the trend of discussion being in that direction from the start.

John (1), on account of his affinity for John Q., lavished the usually deserved compliments upon the author in the usual courteous manner, plus calorified air in this case, because he, John, (1), was captured by a tribe of Hoosier Indians several times and hopes to be again. He said the paper had demonstrated clearly that the fusing of porcelain was a chemical change. This was in the light of a discovery concerning the question whether the change was chemical or merely physical, though Brittanica had it all settled long ago.

John (2) said there was no question at all but that the change was physical, because it was plain to anyone that chemical change could not take place except in the presence of elements subject to chemical change (note the logic). Of course, he said, if those elements were present the change would be physio-chemical. See?

John (1) said, "Well, that's just what I said: the change is chemico-physical."

John (2) then repiled, "That is not the way I understood you. Of course, in the beginning of the process we have hydrogen monoxid present, but if one is at all versed in calory he will understand that it is driven off early in the process. If not, look out for bubbles; but that don't cut any ice in this question of chemical change."

John (1): "How about calor?' Don't that cut any ice? Seems to me the gentleman is getting mixed." (By the way I will explain that calor means heat.)

John (2), replied, "I still contend that my position is right."

John (1) replied to that that John (2) did not fully understand the argument that he, John (1) made. "I simply contend that there is chemical change in fusing porcelain as well as physical."

"So do I," responded John (2). "There is physio-chemical change."

"Well, that's what I have been saying. We agree, but you don't seem to grasp the fact," answered John (1).

Here Dr. Cupidicus butted in and said that he thought both discussants were mixed. He said: "I consider myself conversant with the subject—whatever it is—and calor," he said, "if at the proper altitude, would cause the mix (porcelain) to have a tendency to globulate. This tendency to globularity is a feature of other things than porcelain. The evidence is clear to me every day. I was once a child and they say I was then homely, though of normal proportions. ("Cupid" will have his little joke.) But for many years I have been a hot member of the dental profession, and have become rather rotund. I have given some thought to this chemistry—or whatever it is under discussion—and I know whereof I speak. I know, too, something of hydrogen monoxid on the side, and I agree with the gentleman that it will make bubbles under certain conditions. The essayist knows it, as do other Indians of his tribe, and as many of us know here in our beautiful city by the lake. This fact has been emphasized a good many times and I can not see the use of prolonging the discussion of it. The evidence of what I say is before you. It is as plain as the nose on a man's face."

Here John (3) butted in, and said he knew he was the man alluded at as having demanded evidence of assertions made at a previous meeting by John (1). Said evidence, he said, had been promised but had not yet been forthcoming.

Here John (1) side-stepped by saying he had at different times consulted and advised the essayist, John Q., in his protracted experiments, and the evidence he had to present was embodied in the paper, though under the John Hancock of John Quince. This was the tabulated evidence, but as something more convincing, he drew from his pockets, where he had them concealed, two bricks and threw them—or, rather, threw in a few remarks as he handed them to the audience. They were of porcelain, about as big as beans.

John (3) was by no means convinced and allowed that he knew a whole lot about porcelain that the other Johns did not know, and he didn't propose to enlighten them, either.

John (4) was modest and had to be called out. He made a few remarks pertinent to the subject and returned to his seat.

Then the other Indian from Indian Town, who followed the trail in with John Q., was called upon, and he endorsed some things that John Q. had said, but more particularly what Dr. Cupidicus had said about hydrogen monoxid. He knew more about it since he came to Chicago than before, but, he said, he could put forth only a lame argument, as was indicated by the fact that he was on cruthes three sizes too short for him. An Indian on crutches is one of the symptoms of civilization. He admitted that he was not a real "good Indian," but came near being one under the benign influence of our Lake and John (1).

Some one whose name escaped our stenographer arose at this point and expressed the thought that the audience would be more edified if the discussants confined themselves to the funomia of the paper, and not so much the lake on the side—of our city.

The president said that reference to the lake was pertinent to the subject, as it was largely hydrogen monoxid, and without which we could not work porcelain, nor build a high-ball.

At this a gentleman in the front row sprang to his feet and said he would like to know what all this talk about hydrogen monoxid was. "Every man in this room," he said, "knows enough about chemistry to know that the lake is not hydrogen monoxid, But $H_2O$, pure and simple; and they know, too, that when you heat that up high enough you drive off the $H_2$ and leave the O (Oh!); and if that isn't a chemical change I don't know what is. Let us have common sense!"

Another man said use $C_2H_6O$ and avoid bubbles. I don't know about that, and the essayist didn't enlighten us.

At this point a stranger arose and said the subject had been handled like clay in the potter's hands and much light had been thrown on the difficulties of making inlays and any man who, after listening to the paper and the discussion could not go out and bake inlays without difficulty had better quit the business.

As the argument had been somewhat heated and overdone, a motion to pass the subject was carried and John Quince proceeded to close the discussion; and if John (1), John (2) and John (3) had not interrupted he would have done so.

The meeting—some 300 being present—then adjourned to the College Inn, where the usual banquet provided each month by our president, was partaken, and funomina of baking was forgotten, and hydrogen monoxid+C2H6O, 2½ percent, and malted, was served in steins.—*American Journal.*

